# Stress, coping, and psychological resilience among physicians

**DOI:** 10.1186/s12913-018-3541-8

**Published:** 2018-09-21

**Authors:** Emily O’Dowd, Paul O’Connor, Sinéad Lydon, Orla Mongan, Fergal Connolly, Catherine Diskin, Aoibheann McLoughlin, Louise Rabbitt, Lyle McVicker, Bronwyn Reid-McDermott, Dara Byrne

**Affiliations:** 1 School of Medicine, National University of Ireland Galway, Galway, Ireland; 2 Irish Centre for Applied Patient Safety and Simulation, National University of Ireland Galway, Galway, Ireland; 3 School of Nursing, Midwifery, and Health Systems, University College Dublin, Belfield, Dublin, Ireland; 4 Health Service Executive National Doctors Training and Planning, Dublin, Ireland; 5 Department of Psychiatry, Jonathan Swift Centre, St James’ Hospital, Dublin, Ireland; 6 Department of Medicine, University Hospital Galway, Galway, Co. Galway Ireland

**Keywords:** Psychological resilience, Stress, Coping, Burnout, Workplace challenges, Health service challenges, Patient safety

## Abstract

**Background:**

Recent research has demonstrated that burnout is widespread among physicians, and impacts their wellbeing, and that of patients. Such data have prompted efforts to teach resilience among physicians, but efforts are hampered by a lack of understanding of how physicians experience resilience and stress. This study aimed to contribute to knowledge regarding how physicians define resilience, the challenges posed by workplace stressors, and strategies which enable physicians to cope with these stressors.

**Methods:**

A qualitative approach was adopted, with 68 semi-structured interviews conducted with Irish physicians. Data were analysed using deductive content-analysis.

**Results:**

Five themes emerged from the interviews. The first theme, ‘The Nature of Resilience’ captured participants’ understanding of resilience. Many of the participants considered resilience to be “coping”, rather than “thriving” in instances of adversity. The second theme was ‘Challenges of the Profession’, as participants described workplace stressors which threatened their wellbeing, including long shifts, lack of resources, and heavy workloads. The third theme, ‘Job-related Gratification’, captured aspects of the workplace that support resilience, such as gratification from medical efficacy. ‘Resilience Strategies (Protective Practices)’ summarised coping behaviours that participants considered to be beneficial to their wellbeing, including spending time with family and friends, and the final theme, ‘Resilience Strategies (Attitudes)’, captured attitudes which protected against stress and burnout.

**Conclusions:**

This study emphasised the need for further research the mechanisms of physician coping in the workplace and how we can capitalise on insights into physicians’ experiences of coping with system-level stressors to develop interventions to improve resilience.

**Electronic supplementary material:**

The online version of this article (10.1186/s12913-018-3541-8) contains supplementary material, which is available to authorized users.

## Background

Psychological Resilience (PR) provides protection from workplace stress [1]. Stress has been defined as a physiological and psychological response to perceived threat [2]. In the context of the workplace, it often occurs when an individual perceives the demands of a situation to exceed the resources available to meet these demands [3]. These resources can be organisational, such as staff levels, workload, and adequate pay, or personal, such as self-efficacy, avoidance, and distancing [3]. When resources are exceeded and an individual experiences stress, they can rely on coping mechanisms, which can be either problem-focused (actively changing the stressful environment) or emotion-focused (managing the emotional response to the stressor) [4]. While some research has focused on stress and coping in healthcare workers [2, 3, 5], there is little which explores the mechanisms of this, and how it relates to PR and burnout.

PR has been variously defined as an innate personality trait [6], and as a process of positive adaptation in the face of adversity that can emerge at different life stages depending on the situation [7]. PR has been explored within the context of various professions including social work [8], the military [9], and in healthcare professions such as nursing [10]. However, little research is available that focuses specifically on physicians. A clear understanding of how PR is manifested in doctors, and how to improve or sustain PR in this professional group, is lacking. The absence of PR among doctors has been found to have negative consequences for both physicians and their patients [11, 12]. Typically, interventions designed to improve PR in physicians focus on PR at an individual level, and do not address the organisational issues which impact resilience, such as staffing levels, workload, and lack of resources [13]. However, qualitative research with GPs highlights the need for system-level interventions targeting resilience [14]. Focusing on organisational-level resilience has been identified as important in other areas of research [15]. Research on stress and wellbeing amongst child protection workers has begun to focus on introducing positive, resilience-promoting organisational processes, rather than emphasising individual-centred interventions with beneficial effects [6]. Research on PR among paramedics and firefighters has also underlined the importance and benefits of focusing on organisational factors when examining how resilience can be supported [16, 17]. As the research carried out within these professions has demonstrated, gaining an understanding of how resilience is understood in context of organisational factors can contribute to tackling stress and burnout effectively. Similar research could target improvements in resilience and coping amongst physicians.

Given the emerging evidence indicating benefits of examining resilience in terms of systems level stressors [15–18], it was decided to conduct a qualitative study of physicians to understand the particular challenges faced by doctors to their wellbeing, and how they cope with these, in the context of the health system. The current study aims to: a) understand how a sample of doctors define PR; b) establish what organisational and systems factors serve as stressors and may challenge resilience; and c) learn about the practices and strategies physicians use to maintain their resilience and cope with stressors encountered. It aims to extend the work of Zwack and Schweitzer, a comprehensive study on individual factors relating to resilience amongst physicians [19] by exploring physicians’ perceptions of the challenges to their resilience they face in work and their understanding of what PR looks like, or is, in healthcare settings.

## Methods

This study was conducted in accordance to the CORE-Q reporting guidelines for qualitative studies [20].

### Participants

A total of 68 doctors participated in this research, 39 (57.4%) of whom were female. Interviews were conducted with: 9 Interns; 18 Senior House Officers (SHOs); 18 Specialist Registrars (SpRs); 8 Consultants and 15 General Practitioners (GPs). Specialties included surgery, medicine, anaesthetics, and general practice. Number of years of experience ranged from 0.5 to 33 (mean = 7.19, SD = 7.21, *n* = 68, *n* missing = 2).

### Ethics

Ethical approval was obtained from the Research Ethics Committee at Galway University Hospital. All participants gave consent to participate in the study.

### Interview design

The semi-structured interviews utilised an adapted form of the interview schedule from Zwack and Schweitzer [19]. The purpose of the interviews was to obtain information on: (1) the interviewees’ definition of PR; (2) perceived challenges of their job; (3) strategies they (and others) use to remain resilient and cope with stressors encountered, and; (4) advice they would offer on how to maintain resilience in the challenging context of healthcare environments. The interview schedule, which can be found in Additional File 1, therefore expands upon the Zwack and Schweitzer [19] schedule with the inclusion of questions on defining PR and on the identification of workplace stressors and challenges. These adaptations to the schedule allowed the researchers to gather knowledge essential for the development of effective interventions which incorporate both system- and individual-level factors. The interview schedule was piloted with three doctors in advance of data collection.

### Procedure

The semi-structured interviews were carried out between December 2016 and May 2017. The interviews were carried out by five medical doctors (one male and four females) and two health services researchers (one male and one female). All were trained to carry out the interviews by a psychologist experienced in interview methodologies and used the standardised semi-structured interview protocol.

Participants were recruited using a variety of recruitment strategies. Information on the study was distributed by email and at educational/training sessions. Participants were asked to contact the researchers if they were interested in taking part. After this, a snowball sampling technique was used [21]. On conclusion of the interviews, the interviewees were asked to identify other potential participants. Participants were given a consent form and information sheet prior to the interview. Interviews were conducted either on-site or via telephone. All interviews were audio recorded and transcribed. After transcription, the audio recordings were destroyed. Interviewers summarised the points back to participants, and participants were also offered the opportunity to review their transcript.

Five sample groups were identified and targeted: (1) hospital Consultants (*n* = 8); (2) SpRs (*n* = 18); (3) SHOs (*n* = 18); (4) interns (*n* = 9), and; (5) GPs (*n* = 15). While most participants were stratified by their level of experience, GPs were treated as a separate group given the differences between their role and responsibilities and those of typical hospital doctors. The interviewing continued until data saturation was reached in each of the five groups, and no new themes, subthemes or explanations emerged from the data collected.

### Data analysis

A deductive content analysis approach [22] was used for the data analysis. Two researchers (one male and one female) with PhDs in Psychology performed the coding. The themes and subthemes identified by Zwack and Schweitzer [19] were used as the initial framework for coding the data. The researchers read all the transcripts, coded the interviews against the framework, and compared their coding of the data until consistent coding was achieved. The final coding was then considered and agreed by the researchers. Changes to the Zwack and Schweitzer framework [19] were made through discussion and consensus. These changes included: (1) the theme “Resilience strategies 1: Practices and routines” was rephrased to “Resilience strategies: Protective practices”; (2) some of the descriptors of the subthemes were simplified; and (3) additional themes and subthemes were added.

## Results

A total of 68 interviews were conducted and ranged in length from 3 min 30 s to 34 min 44 s (*M* = 9 min 49 s; *SD* = 6 min 7 s).

### Themes

Five themes emerged from our analysis (discussed below)*.* Each of these themes were further divided into between four and sixteen subthemes. A complete overview of each theme, its component subthemes, and relevant exemplar quotes is provided in Additional File 2.

#### Theme 1: The nature of resilience

**Fig. 1:**
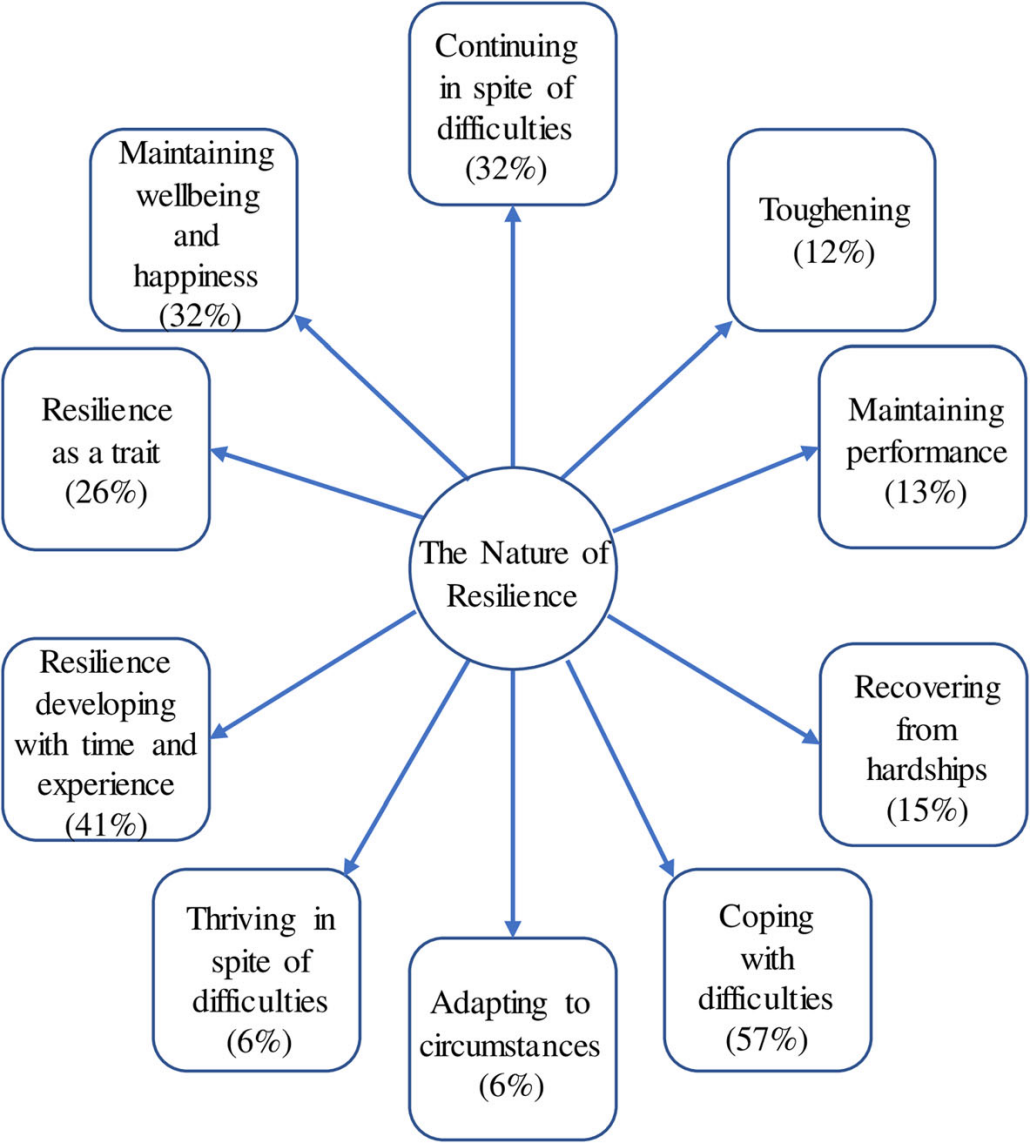
The Nature of Resilience: Subthemes and the percentage of interviews in which they were mentioned

This theme encapsulates how participants defined their understanding of PR during the interviews. This first theme was comprised of eight subthemes (for subthemes, see Fig. 1). ‘Coping with Difficulties’ emerged from 39 interviews, with many of the participants referring to PR as the ability to cope with hardships encountered within the healthcare setting: “*resilience means the ability to endure hardship or stress or arduous periods in work or life*” (SpR 5). Many participants also defined PR as ‘Continuing in Spite of Difficulties’, highlighting their ability to persevere with work despite stressful situations with one GP explaining that *“resilience, I suppose, is your ability to deal with setbacks and bumps in the road without falling apart”* (GP 2). The subtheme ‘Maintaining Wellbeing and Happiness’, in which participants defined PR as keeping a consistent level of wellbeing in the face of stressors, emerged from the interviews with approximately one third of participants. It was commented that PR is comprised of the: “*strategies we develop over the years in order to get by in work and to keep general good mental health*” (SHO 2).

#### Theme 2: Challenges of the profession

**Fig. 2:**
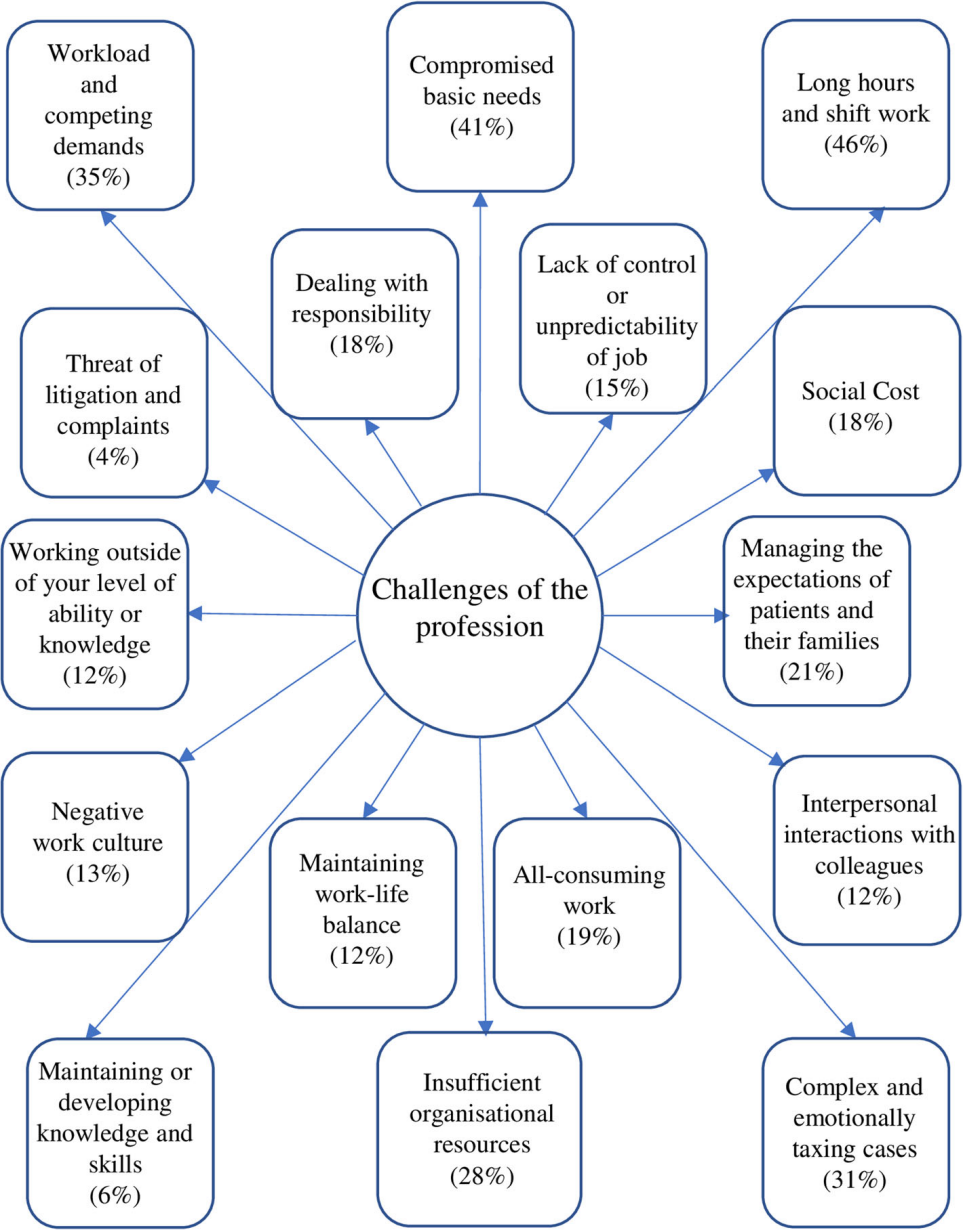
Challenges of the Profession: Subthemes and the percentage of interviews in which they were mentioned

The second theme focused on the stressors and challenges associated specifically with working within the healthcare context that physicians must cope with. Participants spoke extensively on this subject and sixteen subthemes emerged under this heading (see Fig. 2). One subtheme which encapsulated frequently mentioned issues was ‘Long Hours and Shift Work’. The participants described how the number of hours they worked, along with the strain of working unsociable shifts such as the night shift or holidays, threatened their PR. The challenges posed by ‘Long Hours and Shift Work’ were most commonly highlighted by junior doctors: (54% of interns and SHOs; for a breakdown of percentages by specialty, see Table 1) *“I think the most challenging [issue] would be the hours of our work. Sometimes you can be at work for 8 hours, sometimes 12, sometimes 13, and I’ve worked 16 hours a day”* (Intern 1). The subtheme ‘Workload and Competing Demands’ also emerged frequently from interviews with GPs in particular (60% of GPs). They spoke about the difficulties of having multiple high-priority tasks to oversee at the same time, such as seeing lots of patients in a short window of time, and how this heavy workload was challenging to their PR and life outside work. GP9 explained: “*I’m split between three different things at work it’s trying to balance and time manage things that come up…*” A less frequently mentioned (13% of physicians), but interesting subtheme was ‘Negative Work Culture’. This subtheme grouped comments by participants on how the social and cultural aspects of the healthcare system had a significant negative impact on their wellbeing: *“…because of lack of resources, lack of staff et cetera, there’s an increasingly negative vibe or negative working environment where people become quite stressed”* (Consultant 5).

**Table 1:** Number and percentage of interviews which explored subthemes

Theme Subtheme	Whole sample (*n* = 68) *n*; %	GPs (*n* = 15) *n*; %	Interns (*n* = 9) *n*; %	Consultants (*n* = 8) *n*; %	SHOs (*n* = 18) *n*; %	SpRs (*n* = 18) *n*; %
The nature of resilience						
Continuing in spite of difficulties	22; 32%	4; 27%	0; 0%	3; 38%	8; 44%	7; 39%
Maintaining wellbeing and happiness	22; 32%	9; 60%	2; 22%	0; 0%	5; 28%	6; 33%
Toughening	8; 12%	0; 0%	3; 33%	0; 0%	3; 17%	2; 11%
Maintaining performance	9; 13%	1; 7%	1; 11%	3; 38%	2; 11	2; 11%
Recovering from hardships	10; 15%	4; 47%	0; 0%	0; 0%	4; 22%	2; 11%
Coping with difficulties	39; 57%	10; 67%	6; 67%	4; 50%	6; 33%	13; 72%
Adapting to circumstances	4; 6%	0; 0%	1; 11%	0; 0%	2; 11%	1; 6%
Thriving in spite of difficulties	4; 6%	0; 0%	0; 0%	1; 13%	1; 6%	2; 11%
Resilience as a trait	18; 26%	6; 40%	2; 22%	0: 0%	6; 33%	4; 22%
Resilience developing with time and experience	28; 41%	7; 475	5; 56%	1; 13%	8; 44%	7; 39%
Challenges of the profession						
Compromised basic needs	30; 44%	6; 40%	3; 33%	3; 38%	8; 44%	8; 44%
Lack of control or unpredictability of job	10; 15%	4; 27%	1; 11%	1; 13%	2; 11%	2; 11%
Long hours and shift work	31; 46%	5; 33%	2; 22%	1; 13%	13; 72%	10; 55%
Social cost	12; 18%	5; 33%	0; 0%	0; 0%	4; 22%	3; 17%
Managing the expectations of patients and their families	14; 21%	2; 13%	1; 11%	3; 38%	3; 17%	5; 28%
Interpersonal interactions with colleagues	8; 12%	2; 13%	1; 11%	1; 13%	3; 17%	1; 6%
Complex and emotionally taxing cases	19; 28%	5; 33%	3; 33%	2; 25%	3; 17%	6; 33%
All-consuming work	16; 24%	6; 40%	0; 0%	1; 13%	5; 28%	4; 22%
Insufficient organisational resources	18; 26%	8; 53%	0; 0%	5; 63%	2; 11%	3; 17%
Maintaining work-life balance	11; 16%	5; 33%	0; 0%	2; 25%	3; 17%	1; 6%
Maintaining or developing knowledge and skills	4; 6%	1; 7%	1; 11%	1; 13%	0; 0%	1; 6%
Negative work culture	7; 10%	0; 0%	0; 0%	3; 38%	0; 0%	4; 22%
Working outside of your level of ability or knowledge	10; 15%	1; 7%	3; 33%	0; 0%	2; 11%	4; 22%
Threat of litigation and complaints	3; 4%	2; 13%	0; 0%	0; 0%	0; 0%	1; 6%
Dealing with responsibility	12; 18%	8; 53%	1; 11%	0; 0%	0; 0%	3; 17%
Workload and competing demands	24; 35%	9; 60%	3; 33%	3; 38%	4; 22%	5; 28%
Job-related gratification						
Doctor-patient relationship	9; 13%	2; 13%	0; 0%	3; 38%	2; 11%	2; 11%
Medical efficacy	16; 42%	4; 27%	1; 11%	3; 38%	5; 28%	3; 17%
Helping colleagues	5; 7%	2; 13%	0; 0%	2; 25%	1; 6%	0; 0%
Payment	1; 1%	1; 7%	0; 0%	0; 0%	0; 0%	0; 0%
Resilience strategies (protective practices)						
Engage in leisure activities	45; 66%	10; 67%	5; 56%	3; 38%	11; 61%	16; 89%
Support from colleagues	43; 63%	12;80%	5; 56%	5; 63%	10; 55%	11; 61%
Support from family and friends	43; 63%	10; 67%	5; 56%	3; 38%	11; 61%	14; 78%
Know when to ask for help	19; 28%	1; 7%	1; 11%	1; 13%	10; 55%	6; 33%
Maintain boundaries with patients	6; 9%	1; 7%	0; 0%	0; 0%	4; 22%	1; 6%
Prioritisation and delegation	33; 49%	6; 40%	2; 22%	5; 63%	11; 61%	9; 50%
Standing up for yourself	22; 32%	5; 33%	2; 22%	4; 50%	4; 22%	7; 39%
Maintain a professional approach to work	13; 19%	4; 27%	1; 11%	2; 25%	4; 22%	2; 11%
Professional supports	17; 25%	7; 47%	1; 11%	3; 38%	1; 6%	5; 28%
Prioritisation of basic needs at work	8; 12%	2; 13%	1; 11%	1; 13%	2; 11%	2; 11%
Cultivate a good work-life balance	57; 84%	13; 87%	6; 67%	8; 100%	15; 83%	15; 83%
Individual ‘healthful habits’	41; 60%	12; 80%	7; 78%	7; 88%	8; 44%	7; 39%
Resilience strategies (attitudes)						
Acceptance and realism	35; 51%	6; 40%	4; 44%	6; 75%	10; 55%	9; 50%
Self-awareness and reflexivity	41; 60%	12; 80%	4; 44%	3; 38%	9; 50%	13; 72%
Maintaining perspective	48; 71%	9; 60%	7; 78%	5; 63%	16; 89%	11; 61%
Appreciating the good things	5; 7%	2; 13%	1; 11%	0; 0%	1; 6%	1; 6%
Belief in yourself and your abilities	7; 10%	2; 13%	1; 11%	1; 13%	2; 11%	1; 6%

#### Theme 3: Job-related gratification

The theme ‘Job-related Gratification’ captured any aspects of the profession or their role which participants described as being positive, or from which they derived some form of reward, be that emotional or financial. This emerged with less frequency than other themes and has four subthemes (for definitions and examples of these subthemes, see Additional File 2, Table C). ‘The Doctor-Patient Relationship’, in which participants describe their PR benefiting from interpersonal relationships with patients, was discussed by approximately a third of Consultants (Table 1), who highlighted that they derived pleasure or satisfaction from this relationship and these interactions: *“generally having a degree of genuine care for your patients does help your job satisfaction”* (Consultant 6). ‘Medical Efficacy’, the subtheme which captured PR being boosted by helping patients get better, was the only source of gratification mentioned by interns, and related to pleasure they derived from providing effective patient care: “*there is a great satisfaction from the job when you do a good job and there is a good outcome*”.

#### Theme 4: Resilience strategies (protective practices)

Theme 4, “Resilience strategies (protective practices)”, describes behaviours and practices that participants identified as being beneficial to protecting their wellbeing and PR. This theme included 12 subthemes, details of which can be found in Additional File 2, Table D. One example was the subtheme of ‘Cultivate a Good Work-Life Balance’, which was evident within 80% of the interviews conducted (see Table 1). This subtheme captured participants’ descriptions of the importance of removing themselves from the workplace both mentally and physically during their off-time to their PR, with participants saying they: *“try to switch off as much as possible, try not to bring too much home in the evenings if possible”* (GP 13). The subtheme ‘Engage in Leisure Activities’, where participants discussed how activities outside of work can support their PR also emerged frequently- particularly within the subset of junior doctor interviews (56% of interns and 61% of SHOs)- with participants considering it essential to actively engage with recreational activities outside of the workplace. For instance, one intern suggested that it’s important: *“you have a good hobby outside medicine so you don’t end up talking about [work] all day every day”* (Intern 3). ‘Support from Colleagues’, and ‘Support from Family and Friends’, emerged as subthemes from interviews with more than half of participants. These subthemes are related to each, as participants discuss seeking social support and help from either colleagues, or people outside of work, and how this helps them be resilient. Colleagues were considered important as: *“working in a supportive team is a lot different than working in a team where you feel that people don’t have your back. I think key, good relationships, and support structures are important”* (Consultant 7). Relationships outside of work were also considered important to PR: *“family support is a big thing, people who tend to do well are married or in happy relationships and invest properly in those relationships”* (SpR 1).

#### Theme 5: Resilience strategies (attitudes)

The role of particular attitudes or mindsets in promoting or sustaining PR constituted the final theme that was identified from the interviews (see Table 1). This differs from the previous theme (Resilience strategies, protective practices), as while both themes describe strategies participants perceived to maintain, or be integral to resilience, theme four refers to outward behaviour and practices, whereas the present theme relates instead to personal attitudes and thoughts which are considered protective. This theme includes five subthemes (For further info on subthemes, see Additional File 2; Table E). An example of one of the subthemes is ‘Maintaining Perspective’, in which participants described how an attitude towards work which keeps the overall picture in mind, and not letting small things interfere with your wellbeing was an attitude considered to contribute to PR. The protective nature of: “*not taking things to heart…”* was explained by one SHO: *“…nursing colleagues and doctors may say things which, even though they may seem like they are directed at you, are not directed at you”* (SHO 8). In that instance, maintaining perspective involved realising that others are impacted by the system they are working in, and negative interactions with colleagues are not necessarily a result of the physician’s personal actions. Realising that can help maintain PR among physicians. A large proportion of GPs (60%) raised issues relating to the subtheme ‘Self-Awareness and Reflexivity’ which described how PR can be supported through reflective thinking about yourself, holding yourself accountable for your actions, and understanding your role and abilities in each situation. Participants said that *“Those that are not coping well have very little self-awareness, they blame everyone else… They take very little responsibility for the situations [in which] they place themselves”* (GP 10).

A third subtheme, which emerged from interviews with many different groups of physicians, was ‘Acceptance and Realism’, which referred to the fact that within medicine, not everything can go well all of the time. Accepting this and being realistic was described by participants as protective of PR in instances when things do go wrong. This subtheme emerged most frequently from interviews with Consultants (75% of Consultants), with one noting: “*You have to be generous with yourself about mistakes. I think mistakes are always going to happen and you need to look at them objectively, so to divorce the emotion from it and be honest*” (Consultant 3).

## Discussion

The need to understand, and explore means of intervening to improve, physician wellbeing and promote PR in this population is evident [13]. The study reported in this paper sought to contribute to and extend current knowledge regarding how physicians define and understand PR, understand what organisational and systems level factors serve as the primary challenges to PR, and practices or strategies perceived by physicians to promote or sustain PR and cope with workplace stressors. After careful analysis of the data, themes emerged which reflected the complexity of PR and how personal, interpersonal, and systems-level factors interplay to help physicians cope with stressors encountered and remain resilient.

Researchers have frequently noted the difficulties in adopting a definition or consistent conceptualisation of PR [1, 6, 7]. Our study, and particularly the theme ‘The Nature of Resilience’, offers important insights into how physicians understand PR as a construct or attribute in the context of their workplace. Participants primarily viewed PR as individualistic ‘coping’ while encountering continual difficulties and challenges at work. This aligned with some conceptualisations of resilience in the literature, such as that by Britt and colleagues, who explored employee resilience [23]. Britt et al.’s paper challenged the conventional understanding of resilience as thriving or flourishing after experiencing trauma [1] and framed it as employees demonstrating low symptoms of burnout and high wellbeing, maintaining job performance, and having healthy relationships [23]. Other theories of PR, typically within the developmental psychology literature, frame the PR as “flourishing” or growth in adverse or immensely challenging circumstances [1, 24, 25]. However, Britt and colleagues do not consider growth to be necessary for an individual to demonstrate resilience [23]. It is interesting therefore that participants interviewed in the present study viewed PR as coping rather than thriving, which fits with the organizational, rather than the developmental, framework of resilience. This highlights the importance of appreciating the extant understandings of PR within a population prior to introducing any workplace-based intervention targeting resilience. As has been demonstrated previously, a lack of clarity when designing PR interventions can impact negatively on the measurement and evaluation of outcomes [13]. It is therefore important to ensure future interventions consider how PR is defined and experienced by physicians to remain relevant to this population.

Work-related challenges were considered a key impediment to PR for many of the participating physicians, and reflect how it is not only personal characteristics which can influence PR, but also interpersonal relationships with colleagues and the context of the work environment. The idea of work-related challenges impacting on PR overlaps greatly with current research into stress. This research highlights the role played by the workplace in creating stress [26]. These work-related challenges can be considered to be stressors experienced by physicians, manifesting either as an acute, traumatic incident or as a more chronic, continuous strain on their well-being [26]. Some of the stressors mentioned by participants in the present study echoed those experienced by people in other high-stress professions. For example, the subtheme of ‘Complex or Emotionally Taxing Cases’ captures a difficulty also experienced by social workers and clinical psychologists [27–29]. ‘Long hours and shift work’ are a recognised stressor in various sectors such as hospitality and law enforcement [30, 31] and amongst other healthcare staff such as nurses [32]. However, this stressor appears to be particularly pronounced amongst physicians, with 44% of US doctors working more than 60 h per week, compared to an average of 8% of workers across all other professions [33].

‘Insufficient Organisational Resources’, which emerged from our interviews as a work-related stressor, has also been highlighted as clear problem for physicians in previous research, with low staffing and equipment shortages identified as issues [34, 35]. Insufficient organisational resources is reported as a stressor in other professions such as academia [36], indicating that our findings are broadly aligned with multidisciplinary stress research.

One stressor identified by participants that may be unique to physicians is that of the ‘Threat of Litigation and complaints’, an issue heretofore unexplored in relation to PR. It has been found previously that surgeons involved in adverse events suffer emotionally, and are more likely to practice defensive medicine or even stop practicing [37]. This fits with the present study’s finding of litigation as a work-related stressor, and underlines the importance of recognising the complex roles that these stressors play in increasing stress and burnout. A focus on exploring organisational changes to address these stressors alongside offering individual-focused interventions and supports, would contribute to building physician PR and in turn improve overall patient safety [38].

The role of organisational and contextual factors in physician PR was apparent throughout all our interviews. However, as found by Zwack and Schweitzer [19], our participants also emphasised the potential for individual behaviours or practices to influence resilience. Certain practices were considered by our participants to bolster PR and help them cope with work-related stressors, for example, as captured in the subthemes “Cultivating a Good Work-Life Balance”, the related subtheme “Engage in Leisure Activities” and “Support from Family and Friends”. These subthemes have been explored in the literature previously as mechanisms by which physicians cope with the demands of the high-pressure, stressful profession [26]. One Portuguese study identified an association between work-life balance and a lack of resilience leading to the intention to leave work amongst public sector physicians [39]. However, it is clear that many physicians are unable to achieve such balance in their own lives due to conflicts with work and the duties required of them, reflecting how the ability to withstand stress is closely linked to context [40].

Individual behaviours are influenced and impacted by organisational stressors and challenges, reducing and oftentimes negating their benefits [41]. “Support from Family and Friends” was also considered an important coping mechanism by our participants. This is supported by research on how social support impacts wellbeing [42] and reduces stress [43]. Future research should build on the present study by exploring in greater depth the mechanisms through which the above practices improve physician wellbeing and PR, and how to foster these practices to help support PR and reduce stress [44, 45].

In addition to behaviours that may have a positive impact on PR, participants also suggested that certain personal attitudes are intrinsic to maintaining resilience. ‘Self-Awareness and Reflexivity’, along with ‘Belief in Yourself and your Abilities’ when working in stressful situations, were both mentioned as important factors in protecting PR by our participants. Research in palliative medicine has previously demonstrated how self-awareness can predict a physician’s ability to cope with patient death and their propensity to burnout [46], which supports the emergence of this theme from our study. ‘Belief in Yourself and your abilities’, or having high levels of self-efficacy has been shown to protect against stress and burnout in the nursing literature [47]. The extent to which our study resonates with previous literature indicates that protective attitudes are central to combating the organisational stress faced by physicians and must be considered when examining the mechanisms by which doctors remain resilient. This knowledge, in turn, could inform resilience interventions which take into account physician and system-level factors, and improve on interventions which have previously shown to be relatively poor [13].

### Recommendations

Our data suggest several important recommendations concerning the study and promotion of PR among physicians, to protect against the pervasive stress of the healthcare setting, going forward. First, techniques to build PR at an individual level are warranted, as physicians report drawing on personal practices and attitudes to remain resilient in this and other studies [19, 48]. However, a focus on developing interventions with a systems-level approach that address workplace stressors and challenges (e.g., long hours and shift work, lack of resources, workload) are essential to complement and support interventions focused on the individual [13, 49].

Second, given that the themes which emerged from the interviews, while centred on PR, were so closely linked to concepts of stress and coping, it would be interesting to examine the relationships between these concepts in a quantitative fashion. Conducting both quantitative and qualitative investigations on the same concepts can ensure reproducible, externally valid findings which are based on the lived experience of participants, leading to more grounded conclusions on the topic [50].

Third, although previous research has highlighted the difficulties around defining and measuring PR [1, 6, 7], physicians in the current study were remarkably consistent in their understanding of the concept. Existing literature has emphasised that measurement of resilience to-date is of variable quality [13], and that there is a need to improve the validity of measurement processes [13], potentially through the development of physician-specific measurement instruments [13]. The data provided in this paper, which offer a comprehensive insight into how resilience is defined, workplace stressors in the healthcare context, and behaviours and attitudes perceived to be related to resilience may be useful for informing the development and content of such an instrument.

### Limitations

There were certain limitations to this study that should be acknowledged. First, the study used the framework set out by Zwack and Schweitzer in their 2013 paper [19] as a starting point for the research. Some might argue this decision was a limitation as it somewhat dictated the direction in which the study would progress. However, this framework was used as the basis of the study as the initial study comprised the large, most comprehensive consideration of physician resilience to-date. The current study sought to extend and progress their data, and capitalising on existing data was considered the best way to advance knowledge in the field. As a result, we have learned that similar themes have emerged from a sample in a different country, and in the context of a different health system which emphasises the external validity of our findings. Second, this study employed a qualitative research design; therefore, generalisability is not guaranteed [51]. While further quantitative work in this area is certainly essential to explore the generalisability of the findings, qualitative research provides a depth of understanding into physician experience which can complement and enrich numerical data [52]. Next, a deductive approach was used for the analysis, which can be considered a limitation in that it could potentially bias the analysis, as researchers enter the analysis stage with preconceptions surrounding the data [53]. However, it is a well-established method for analysis when a theory already exists, and makes explicit the fact that researchers are unlikely to be entering into analysis naïvely [53]. Therefore, it was an appropriate method of analysis for the present study, which aimed to extend the work of Zwack and Schweitzer [19] and truly advance knowledge in this research area. Finally, while the sample size was relatively large, the duration of the interviews was typically short, with a mean of under 10 min due to constraints on participants’ time. While this limited our ability to explore to a greater depth the lived experiences of our participants, they were still able to provide us with insights into physician resilience, and factors that detract and/or contribute to it within the inherently stressful healthcare environment.

## Conclusions

This study explored physicians’ understanding and experiences of PR, along with their perceptions of work related stressors and coping strategies used to maintain resilience and manage stress. In our sample, physicians conceptualised PR as “coping”, which differs from some conceptualisations of PR as “thriving” in difficult situations, although echoes and extends other findings within a workplace context. While the participating physicians readily identified personal attitudes and behaviours that contribute to supporting them in their stressful workplace, they also emphasised the role that workplace stressors play in compromising PR and engendering feelings of burnout. These findings have emphasised a) the need for further research regarding how PR can be improved in this population; b) that the concepts of stress, burnout, resilience and coping appear to be very much related in the context of healthcare; and c) how interventions to improve physician coping in the workplace must capitalise on insights into physicians’ experiences, particularly regarding the influence of system-level factors on resilience.

## Additional files

Additional File 1: Interview Schedule. This file includes the interview schedule used by the researchers when conductin the 68 interviews in the current study. (PDF 188 kb)

Additional File 2: Themes and subthemes identified, along with exemplar quotes. This file presents tables of the themes and subthemes that emerged after analysis of the data collected in this study. Exemplar quotes for each of these themes and subthemes are also presented. (PDF 198 kb)
