# Supplementary material for: Stress, coping, and psychological resilience among physicians
Source: BMC Health Serv Res. 2018 Sep 21;18:730. doi: 10.1186/s12913-018-3541-8 (PMC6151040; doi:10.1186/s12913-018-3541-8)
Supplement: Supplementary file 1 — Interview Schedule. This file includes the interview schedule used by the researchers when conductin the 68 interviews in the current study. (PDF 188 kb) [file 12913_2018_3541_MOESM1_ESM.pdf]

Additional File 1. Interview Schedule

Interview Questions
General Demographic Data: Professional experience, grade, specialty
What does resilience mean to you?
Which are the major challenges of your profession to your mental and physical health and job satisfaction? How successful would you consider your personal coping?
How can a physician remain healthy and satisfied? What strategies do you apply? Are these the most important resilience strategies in your professional life?
Of those colleagues that you would deem to have the greatest levels of resilience, what strategies do they apply in order to deal with the stressors of professional life?
Of those colleagues that you would deem to have the lowest levels of resilience, what strategies do they fail to apply to deal with the stressors of professional life?
If a medical student asked you what he/she could do to increase resilience: Which advice would you give? What mistakes you made yourself would you warn against?
Is there anything else we should have asked?
