# Supplementary material for: Stress, coping, and psychological resilience among physicians
Source: BMC Health Serv Res. 2018 Sep 21;18:730. doi: 10.1186/s12913-018-3541-8 (PMC6151040; doi:10.1186/s12913-018-3541-8)
Supplement: Supplementary file 2 — Themes and subthemes identified, along with exemplar quotes. This file presents tables of the themes and subthemes that emerged after analysis of the data collected in this study. Exemplar quotes for each of these themes and subthemes are also presented. (PDF 198 kb) [file 12913_2018_3541_MOESM2_ESM.pdf]

Additional File 2. Themes and subthemes identified, along with definitions and exemplar quotes.

Table A. The Nature of Resilience

Subtheme	Definition	Sample quote
Continuing in Spite of Difficulties	PR as the ability to persevere with work despite challenges faced within the workplace	"The ability to endure and persist during difficult times" SPR 18
Maintaining Wellbeing and Happiness	PR as keeping a consistent level of happiness and wellbeing in the face of stressors	"What strategies we develop over the years in order to get by in work and to keep general good mental health" SHO 2
Toughening	PR as hardening to withstand stressors in the workplace	"Resilience to me would imply a level of toughness" SHO 16
Maintaining Performance	PR as continuing to perform at work despite challenges	"Persistence in establishing goals and overcoming any obstacles in path to pursuit of goals" Consultant 1
Recovering from Hardships	PR as a process of recovery from a stressful event	"Resilience is your ability to pick yourself up when you are faced with adverse circumstances…" GP 8
Coping with Difficulties	PR as using coping mechanisms to deal with adverse events	"The ability to be flexible and cope with a stressor with something that either be mentally or physically or spiritually challenging" Intern 1
Adapting to Circumstances	PR as a process of adjusting to workplace events to reduce stress	"To adapt to circumstances without it affecting yourself" SHO 12

Thriving in Spite of Difficulties	PR as performing well despite adverse circumstances	"Resilience means thriving in the face of adversity" SHO 13
Resilience as a Trait	PR defined as something which is inherent, a steady personality trait	"Maybe if you think that it's something that you can pick up. I don't think you can, I think if they're a person that take things to heart, it's not something that you can give people" SHO 7
Resilience Developing with Time or Experience	PR as something that can be learned or developed through experience	"And then I think sometimes it's just experience, 2 years in versus 10 years in you look at things very differently" SPR6

Note: GP=General Practitioner, SHO=Senior House Officer, SpR=Specialist Registrar

Table B. Challenges of the Profession

Subtheme	Definition	Sample quote
Compromised Basic Needs	Basic needs such as food, rest, comfort breaks compromised at work	"… you were expected to work non-stop, you were only allowed to take breaks when somebody else decided you were allowed to take breaks as if you were in a prisoner of war camp or something" Consultant 8
Lack of Control or Unpredictability of Job	Having little personal control over the workday	"A big thing would be the loss of control in your day, there's no strict start time or end time…" Intern 4
Long Hours and Shift Work	Working more than the average, and working unsociable shift hours	"The biggest challenge were the hours. I'm not someone who functions well without sleep…" SPR 1
Social Cost	Work threatening social and relationship aspects of life	"I think moving as well, so you're moving around every year for… your career until you get settled somewhere its incredibly difficult" SPR 6
Managing the Expectations of Patients and their Families	Dealing with unrealistic hopes and expectations from patients as a stressor or challenge	"I suppose the patient expectations put on you to fix your problems and the expectation you put on your yourself that you should be able to fix people's problems for them" SPR 19
Interpersonal Interactions with Colleagues	Challenging relationships with the team in work threatening resilience	"Lack of teamwork between MDT people. Sometimes the lack of teamwork between nurses and doctors would be a barrier…" SHO 6
Complex or Emotionally Taxing	Upsetting or stressful cases taking	"I look after patients with a history of sexual assault so I hear some

Cases	a toll on physicians' resilience	horrible stories which can get in on you so you have to have measures to deal with it" Consultant 3
All-Consuming work	Not being able to switch off from work/detach from emotionally draining aspects	"Those who have bigger hearts, those who are more emotionally involved with patient groups… if you are that way inclined I think you will suffer more and tend to burn out quicker" GP 7
Insufficient Organisational Resources	Lack of physical resources from an organisational level	"The inability to get service improvement or just the provision at a proper level. Resource-poor and not being communicated with". Consultant 3
Maintaining Work-Life Balance	Work interfering with other aspects of life/taking time from the rest of life	"I don't live within the practice area so I don't have too many patients calling to the door, but I do have some out-of practice stuff, which does take up a lot of time… through email it certainly has impacted on quality of home life" GP 11
Maintaining or Developing Knowledge and Skills	Difficulty keeping up with developments in the field	"Constantly having to learn new things and being unfamiliar with certain things can be quite taxing, it can be quite exasperating to feel that you have something else to learn, there's always something unfamiliar that will crop up." Intern 7
Negative Work Culture	Social and cultural atmosphere in health system is negative	"You do put up with a lot of verbal abuse. Not necessarily said but with bad intentions, but it is a hyper masculine environment." SPR 5
Working Outside of your Level of Ability or Knowledge	Stress of being out of your depth in a work situation	"Situations where your Reg and your SHO is off and you're post-call and then you're in a situation where you have to do something where you're not really comfortable in doing but there's not much you can do

		to ask for help." Intern 3
Threat of Litigation and Complaints	Challenge of dealing with potential legal action and complaints from patients	"[the biggest challenge] For myself would be the threat of litigation which I dealt with during my intern year." SPR 17
Dealing with Responsibility	Stress of having responsibility for care of patients	"you're responsible for everything that happens to patients as you're co-ordinator and diagnostician for all of their health" GP 4
Workload and Competing Demands	Challenge of having multiple tasks to prioritise and a heavy workload	"I guess I suppose work volume increase year on year without increasing resources, so an ever increasing workload is the biggest thing and I would say prioritising and managing that workload without becoming overwhelmed" Consultant 5

Table C. Job related gratification

Subtheme	Definition	Sample quote
Doctor-Patient Relationship	Gaining gratification from interpersonal relationships with patients	“Generally having a degree of genuine care for your patients does help your job satisfaction as well as you’re not just doing it for a paycheck, you’re working for the genuine interest for your patient” Consultant 6
Medical Efficacy	Feeling gratified from helping patients get better	“Providing the best care to your patients is best for yourself, at the end of the day, to feel like I have accomplished something.” SPR 5
Helping Colleagues	Gratification from helping colleagues deal with stressful situations	“It’s also talking with colleagues and saying “Oh, that must be tough”, they haven’t taken a thing off you, all they’ve done is acknowledge it, but by acknowledging it, they’ve almost made you feel like someone else feels my pain here…” GP 10
Payment	Financial remuneration as a benefit of the job	“You can kind of put up with everything that’s thrown at you, the long hours and hard work better when you’re adequately remunerated” GP 7

Table D. Resilience Strategies: Protective Practices

Subtheme		Sample quote
Engage in Leisure Activities	Use of activities outside of work to boost PR	"I usually try to have some sort of holiday planned, not too often, but I do try to have something planned and something to look forward to." SHO 10
Support from Colleagues	PR supported through seeking help from co-workers	"I think it's good to hang out with people who are in the job, who you can kind of….vent about work with and they understand. It's different when you talk to people who aren't in the job because they don't really get it." SHO 7
Support from Family and Friends	PR maintained by seeking social support from people outside of work	"I would say maintaining good friendships outside work and an active social life" GP 14
Know When to Ask for Help	Asking for help when out of your depth protective of PR	"Don't be afraid to say what happened if you did something wrong. It's better to talk about it because every intern has these experiences" Intern 3
Maintain Boundaries with Patients	Actively keeping a distance from patients personally to protect PR	"You have to realise that you're not your life, while at the same time treat it with importance you have to have a systematic approach to everything." SHO 11
Prioritisation and Delegation	Planning and prioritising time is supportive of PR	"I think time management and prioritising is probably the most important … I know there are things you can't plan for as well but…it's about being aware what's coming up" GP 12
Standing up for Yourself	Defending yourself against patients in stressful situations	"I think the majority have very good boundaries, I think they'll accept what they can accept when services are very limited they will say I cannot

	seen as protective	provide that service…" Consultant 6
Maintain a Professional Approach to Work	Behaving professionally at work seen as protective of PR	"Another thing as well I suppose is just building competency in your job, I know as I have gotten more senior I get less stressed out in the hospital." SPR 1
Professional Supports	Accessing official supports for physicians to protect PR	"But there is some role for coaching. I did a diploma in coaching and I feel there is a real role for students and other people to received coaching." Consultant 6
Prioritisation of Basic Needs at Work	Making time in work to look after your own needs protects PR	"Eating regularly is a big thing. If things aren't important and I need to eat I'll just go eat." Int 4
Cultivating a Good Work-Life Balance	Making an effort to remove yourself from workplace mentally and physically when not on duty in order to protect PR	"Detaching it, detaching it from work so if it is your day off, you're off and not coming in for other things." GP 12
Individual 'Healthful Habits'	Taking part in healthy activities such as mindfulness, healthy eating, exercise	"So I'm a daily meditator with my husband, I would've done a Mindfulness Based Stress Reduction program. …It's been really helpful and…I now sort of nourish mindfulness and wellness" Consultant 7

Table E. Resilience Strategies: Attitudes

Subtheme	Definition	Sample quote
Acceptance and Realism	Supporting resilience by having a realistic attitude to work, and accepting negatives	"I think the people with the greatest levels of resilience are those with the most realistic outlook on what they're dealing with…" SHO 11
Self-Awareness and Reflexivity	PR supported by knowing your own self and being able to reflect on this	"Knowing yourself, knowing your competency or on an emotional level is important." SPR 1
Maintaining Perspective	PR supported by keeping perspective on situations	"I suppose look at it in the greater picture and not take the burden on your shoulders" Consultant 1
Appreciating the Good Things	Supporting PR through acknowledging and appreciating good aspects of the job	"So my advice would be to try and accept your circumstances and work with them and not to get frustrated by them and just make the best of the good things that are there." Int 10
Belief in Yourself and your Abilities	Self-efficacy and belief in skills boosting PR	"It's work, so you've learnt the skills, you know what to do so be,…don't doubt yourself. Even if you have some doubt, don't doubt yourself" SHO 15
